# Psychological Strain and Suicidal Ideation in Athletes: The Multiple Mediating Effects of Hopelessness and Depression

**DOI:** 10.3390/ijerph17218087

**Published:** 2020-11-02

**Authors:** Guoxiao Sun, Jingyi Zhao, Siyu Tian, Liwei Zhang, Cunxian Jia

**Affiliations:** 1 School of Physical Education, Shandong University, Jinan 250061, China; sunguoxiao@sdu.edu.cn (G.S.); ZhaojingyiSports@mail.sdu.edu.cn (J.Z.); tiansiyu@mail.sdu.edu.cn (S.T.); 2 Department of Epidemiology, School of Public Health, Cheeloo College of Medicine, Shandong University, Jinan 250012, China; 3 School of Psychology, Beijing Sport University, Beijing 100084, China; liweizhang@hotmail.com; 4 Center for Suicide Prevention Research, Shandong University, Jinan 250012, China

**Keywords:** mental health, athletes, suicidal ideation, psychological strain, hopelessness, depression

## Abstract

The present study aims to examine the relationship between psychological strain, suicidal ideation, hopelessness, and depression among Chinese athletes. Participants were 774 Chinese athletes (454 men and 320 women), with a range of ages from 12 to 35 (*M* = 18.47, *SD* = 3.39). The structural equation modeling method was used to examine the multiple mediating effects of hopelessness and depression between psychological strain and suicidal ideation. As expected, a positive correlation between psychological strain, suicidal ideation, hopelessness, and depression was found. Additionally, results seem to indicate that psychological strain directly and positively influenced suicidal ideation, and that hopelessness and depression played a serial mediating role in the relationship between psychological strain and suicidal ideation. To conclude, the psychological strain theory is applicable for explaining suicidal ideation in athletes. In athletes, psychological strain is sequentially associated first with a sense of hopelessness and then depression, which is in turn related to suicidal ideation. The present study makes a significant contribution to the literature because we provide a new theoretical basis and new methods for preventing mental disorder and suicidality in athletes.

## 1. Introduction

Research on the mental health of athletes has grown rapidly in recent years [1,2]. Several studies have shown that athletes often demonstrate serious mental health problems [3,4,5,6]. Athletes often face unprecedented crises, difficulties, and challenges in their professional lives, which may combine to increase the risk of developing mental health problems [7]. Rao and Hong suggested that further study is required to understand and promote athletes’ mental health [8].

Examining and clarifying the problem of suicidal ideation in athletes is necessary to alert academic, social, and governmental departments to the mental health issues of athletes. Research on suicidal ideation among athletes will help to uncover the conditions and needs of athletes when they are in abnormal psychological states, help others provide better support, and help athletes to cope better with difficult situations [9]. Suicidal ideation is a high-risk factor of suicidal behaviors [10,11]. Studying suicidal ideation and its risk factors can help screen out potential high-risk groups, so as to allow for interventions prior to the formation of suicidal ideation and prevent the occurrence of suicidal behaviors [12].

Suicidal ideation and behaviors have been regarded to be influenced by various biological, social, and psychological factors [13]. Psychological strain theory has been proposed to explain suicide [14,15]. Unlike a simple stress, the strain consists of at least two conflicting stressors, which pull or push an individual in different directions [14]. Psychological strain is therefore much more devastating to an individual’s wellbeing than a single stress. Psychological strain is much like cognitive dissonance, but in terms of psychological impact, it can be more powerful, serious, and threatening [15]. The strain theory posits that suicide is usually preceded by psychological strains [15]. The theory consists of four major aspects: (1) value strain, due to two or more conflicting internalized values; (2) aspiration strain, due to obvious discrepancy between one’s expectation and reality; (3) deprivation strain, due to relative poverty or deprivation; and (4) coping strain, experienced when an individual is confronted with a crisis but feels unable to deal with it properly [14]. Four sources of strain cause psychological frustration, which progressively leads to suicidal behavior through the mediation of social, psychological, and psychopathological factors [15].

The strain theory has been verified in samples such as college students, Chinese rural young people, and so on [16,17]. The instrument of measuring psychological strain, the Psychological Strain Scale, has also been proven to have good reliability and validity [18,19]. However, whether the strain theory is applicable to athletes remains to be investigated. Athletes often encounter challenges, difficulties, or crises in their career, which may occur much more frequently than for the general population, and that might lead to psychological strain [20]. On the other hand, athletes who grow up in adversity might have developed better resilience to cope with stressful situations [21]. Therefore, whether psychological strain is associated with suicidal ideation in athletes needs to be tested.

There is much evidence that psychological strain relates to suicidal ideation [16,22], but there remains a relative lack of studies investigating the mechanisms underlying this relationship. Hopelessness, which is characterized by pessimistic expectations for the future, lack of general motivation, and the attribution of negative meanings to personal experiences, has been reported to be associated with increased risk of suicide [23,24,25,26,27]. In addition, Zhang et al. revealed that hopelessness mediates the relationship between psychological strain and suicidal ideation among patients diagnosed with stomach cancer [28]. Hopelessness may occur when athletes feel that there is no way to deal with conflicting situations. For some athletes, suicide may become a final solution to relieve the feeling of hopelessness [29].

Meanwhile, Zhang and Lyu found that psychological strain demonstrates a strong correlation with depression [19]. In addition, Stranieri and Carabetta reported that depression and suicide are closely linked [30]. Depression was also reported to be a risk factor for suicide among athletes [31]. It is worth further investigating whether depression mediates the relationship between psychological strain and suicidal ideation among athletes. This exploration will contribute to a better understanding of the psychopathology mechanism underpinning suicide [32].

Hopelessness has been regarded as a factor influencing depression [33]. According to the hopelessness theory of depression, hopelessness is considered to be a proximal cause of depression [34]. Mac Giollabhui et al. revealed that hopelessness mediated the impact of the interaction of a negative life event and negative cognitive style on depression, thus verifying the hopelessness theory of depression [35]. A negative life event combined with negative cognitive style (i.e., negative coping style) may lead to psychological strain. It is therefore predicted that hopelessness mediates the relationship between psychological strain and depression. Waszczuk et al. further revealed that hopelessness affected depression not only cross-sectionally but also longitudinally [36]. Efi et al. also found that depression played a mediating role in the relationship between hopelessness and desires for hastened death among patients with advanced cancer [37]. Based on the previous findings, we assumed that hopelessness and depression play serial mediating roles between psychological strain and suicidal ideation. Athletes who are confronted with conflicting stress are more likely to have a sense of hopelessness for the future before feeling depressed, and eventually generate a thought of committing suicide.

The purpose of this study was to introduce psychological strain theory to explain suicidal ideation and investigate the direct and indirect effects of psychological strain on suicidal ideation in athletes. We expect (1) psychological strain to significantly affect suicidal ideation in athletes; (2) hopelessness and depression to separately mediate the relationship between psychological strain and suicidal ideation; and (3) hopelessness and depression to play a serial mediating role between psychological strain and suicidal ideation.

## 2. Methods

### 2.1. Participants

A total of 807 athletes (467 male and 340 female) who were currently competing at international, national, or provincial levels were recruited. We deleted the data of 32 athletes who were younger than 12 years old and removed data of one basketball player who was at the age of 49. The final sample size used for analysis was 774. The mean age was 18.47 years (*SD* = 3.39), with a range of 12 to 35 years, including 412 adolescents (from 12 to 18 years) and 362 young adults (from 19 to 35 years). Among them, 24 were international-level athletes (top 8 in the Olympic Games or top 6 in World Championships), 96 were top 3-level in national competitions, and 259 were top 8-level in national competitions. These athletes were defined as elite athletes in the current study.

We conveniently selected three Chinese national and twenty-four provincial sports teams, covering confrontational sports, such as taekwondo, judo, tennis, football, basketball, volleyball, and non-confrontational sports, such as gymnastics, trampoline, wushu, shooting, archery, ski, swimming, track and field, and so on. Participating athletes completed the questionnaires via an online platform *Questionnaire Star* (https://www.wjx.cn/). The survey package was distributed via an online link in the team meeting where the anonymous and confidential nature of the survey was explained. The instructions were given by the coaches who had been guided to give uniform instructions to participating athletes. This study was approved by the Ethics Committee of Shandong University School of Public Heath (No. 20190609); all participants were fully informed about the purpose and methodology of the study and were granted the right to agree or refuse to participate before filling in the questionnaires.

### 2.2. Measures

#### 2.2.1. Demographic Variables

The demographic information collected included age, gender (male/female), sports level (elite/non-elite), sports event (confrontational/non-confrontational), residence (urban/rural), and whether the participant was an only child or not.

#### 2.2.2. Psychological Strain Scale (PSS)

To measure psychological strain, the Psychological Strain Scale (PSS) was used [18]. PSS comprises four subscales: value strain, aspiration strain, deprivation strain, and coping strain. Each of the four dimensions was measured by 15 items. Five items from the value strain subscale were deleted because they were not considered suitable for Chinese athletes. A five-point Likert scale was used to measure responses, with “1” representing strongly disagree and “5” representing strongly agree. The responses to each of the respective 15 items were averaged to calculate the score of each dimension. The score of each dimension was averaged to calculate the strain value. The higher the value, the greater the psychological strain. Cronbach’s α coefficient was 0.972 for the total scale, 0.860 for the value strain subscale, 0.931 for the aspiration strain subscale, 0.931 for the deprivation strain subscale, and 0.925 for the coping strain subscale.

#### 2.2.3. Beck’s Hopelessness Scale (BHS)

To measure hopelessness, we used the Beck’s Hopelessness Scale (BHS) [38]. BHS comprises 20 items including 10 negatively formulated items. Participants were required to report “yes” or “no” on the statement of each item. Scores for the 20 items ranged from 0 to 20, with higher scores denoting a more severe sense of hopelessness. Cronbach’s α coefficient was 0.783 for the total scale, revealing acceptable reliability.

#### 2.2.4. The Center for Epidemiologic Studies Depression Scale (CES-D)

To measure depression, we used the Center for Epidemiologic Studies Depression Scale (CES-D), which is applicable for measuring depression in non-clinical populations [39]. Studies have shown that the reliability and validity of this scale are good [40]. Participants were asked to report how frequently they had experienced each symptom. A 4-point Likert scale was used, with “0” representing less than a day, “1” representing 1–2 days, “2” representing 3–4 days, and “3” representing 5–7 days. Four negatively formulated items were reversed before summing the scores. Scores for the 20 items ranged from 0 to 60, with higher scores denoting more severe depressive symptoms. Cronbach’s α coefficient was 0.809 for the total scale, revealing acceptable reliability.

#### 2.2.5. Suicidal Ideation

There are two ways to measure suicidal ideation. One is to use the suicide ideation scales, but owing to the large number of items in the scale, the expression of the question is too direct, leading to rejection. Another method is to use a single item for measurement, which is more commonly used [26,41]. In this study, suicidal ideation was measured using one item: “Have you ever thought of killing yourself?” A total of 18% athletes reported “yes”.

### 2.3. Statistical Analysis

The original data in Excel format was exported from the Questionnaire Star (https://www.wjx.cn/) platform and checked to ensure that there were no invalid questionnaires. SPSS 24.0 (IBM, Armonk, NY, USA) was employed to obtain descriptive statistics, correlation values, and multivariate logistic regression results. AMOS 24.0 (IBM, Armonk, NY, USA) was used for the multiple mediation analysis. A structural equation model (SEM) was constructed to examine the theoretically indicated hypotheses, using a maximum-likelihood (ML) estimator. The model was considered to be a good fit based on the following criteria: the chi-square test, comparative fit index (CFI > 0.90), Tucker–Lewis index (TLI > 0.90), normed fit index (NFI > 0.90), relative fit index (RFI > 0.90), and root mean square error of approximation (RMSEA < 0.08) [42]. A bootstrap method (sampling was repeated for 2000 times) was adopted to construct 95% confidence intervals (CIs) for significance testing of the indirect effects. An indirect effect is considered significant when the 95% CI does not include zero.

## 3. Results

### 3.1. Common Method Bias Testing

Common variance analysis was applied to the four questionnaires through factor analysis. The chi-square statistic of Bartlett’s test of sphericity was significant (KMO = 0.951, *p* < 0.001). After principal component analysis, 16 eigenvalues greater than 1 were extracted. The first factor to explain the variance was 25.36%, which was less than the 40% required by critical standards, demonstrating that the questionnaires used in the current study had no significant issue with common method biases [43].

### 3.2. Demographic Variables in the Sample

Among the 774 athletes, 454 (58.66%) were males, 379 (48.97%) were at the elite level, 320 (41.34%) were from confrontational sports, 376 (48.58%) were from rural areas, and 331 (42.76%) were from only-child families.

### 3.3. Correlation Analysis

Means, standard deviations, and a correlation matrix for psychological strain, hopelessness, depression, and suicidal ideation are presented in Table 1. A bivariate correlation analysis showed that the variables were significantly and positively correlated with each other (*p* < 0.01).

### 3.4. The Association of Demographic Variables with Psychological Strain, Suicidal Ideation, Hopelessness, and Depression

We analyzed the association of demographic variables with psychological strain, suicidal ideation, hopelessness, and depression (Table 2). Age was found to be positively correlated with hopelessness and depression. Female athletes demonstrated greater value strain and suicidal ideation, but lower hopelessness. Elite athletes demonstrated greater value strain, coping strain, suicidal ideation, and hopelessness. Athletes playing non-confrontational sports demonstrated higher levels of psychological strain, hopelessness, and depression. Athletes who were from urban areas and only-child families demonstrated higher levels of hopelessness and depression.

### 3.5. Risk of Suicidal Ideation among Athletes by Multivariate Logistic Regression Analysis

A multivariate logistic regression analysis was performed to identify the risk factors for suicidal ideation among athletes (Table 3). Demographic variables, psychological strain, hopelessness, and depression were entered stepwisely. As the four strains showed medium to high correlation (see Table 1), we entered the global strain dimension instead of all four dimensions so as to avoid the issue of collinearity. The results showed that younger athletes (odds ratio OR = 0.913, *p* = 0.008), elite athletes (OR = 3.122, *p* < 0.001), and individuals who had higher levels of depression (OR = 1.065, *p* < 0.001) were more likely to have suicidal ideation. Psychological strain and hopelessness influenced suicidal ideation, but the effects disappeared after depression was entered, suggesting that depression mediated the effects of psychological strain on suicidal ideation, and hopelessness on suicidal ideation.

### 3.6. Mediation Analysis

We constructed and examined the serial mediating effect of hopelessness and depression between psychological strain and suicidal ideation. The data fit the model well (χ^2^ = 52.608, df = 13, χ^2^/df = 4.047, CFI = 0.995, TLI = 0.990, NFI = 0.994, RFI = 0.986, RMSEA = 0.063). However, we noticed that the path from hopelessness to suicidal ideation was not significant (β = −0.074, *p* = 0.094) and thus deleted this path. The data fit the modified model well (χ^2^ = 55.403, df = 14, χ^2^/df = 3.957, CFI = 0.995, TLI = 0.990, NFI = 0.993, RFI = 0.987, RMSEA = 0.062).

Table 4 presents the results of the path analysis of the four variables and shows the standardized coefficients, their standard errors (*SE*s), 95% confidential intervals (CIs), and *p* values. The effect of psychological strain on suicidal ideation was significant (β = 0.087, *p* = 0.026), indicating that psychological strain may be a direct factor leading to suicidal ideation among athletes. Psychological strain was significantly and positively associated with hopelessness (β = 0.251, *p* = 0.001), and depression (β = 0.282, *p* = 0.001), suggesting that athletes who have greater psychological strain are more likely to feel hopeless and depressed. Similarly, hopelessness had a positive impact on depression (β = 0.543, *p* = 0.001). In addition, the association between depression and suicidal ideation was positive (β = 0.201, *p* = 0.001).

**Figure 1:**
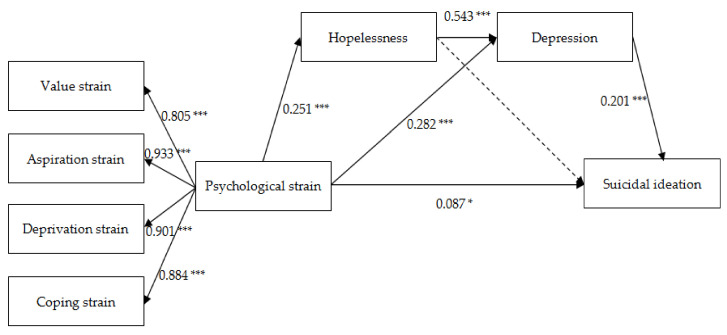
Serial mediating effect of hopelessness and depression between psychological strain and suicidal ideation; * *p* < 0.05; *** *p* < 0.001.

As shown in Figure 1, psychological strain was significantly associated with hopelessness, which was in turn correlated with depression and eventually contributed to suicidal ideation, indicating that hopelessness and depression played a serial mediating effect between psychological strain and suicidal ideation among athletes. Additionally, there was a sole mediating effect of depression on the relationship between psychological strain and suicidal ideation.

Mediation analysis based on the bias-corrected bootstrap method with 2000 samples was conducted to estimate the indirect effects of psychological strain on suicidal ideation mediated by hopelessness and depression. The effect sizes, their standard errors, and 95% CIs of the direct, indirect, and total effects are illustrated in Table 5. The direct effect of psychological strain on suicidal ideation was significant (95% CI: 0.011–0.164), and the total indirect effect was also significant (95% CI: 0.052–0.120). The indirect effects of psychological strain on suicidal ideation via the mediation of depression (95% CI: 0.035–0.082) and hopelessness and depression (95% CI: 0.016–0.042) were both significant.

## 4. Discussion

Suicidal ideation among athletes is worthy of attention [9]. In this study, the percentage of athletes with suicidal ideation was 18%, revealing the significance of this problem. We introduced psychological strain theory to investigate suicidal ideation among athletes [15]. Our major findings were as follows: first, psychological strain was associated with suicidal ideation in athletes; second, hopelessness and depression played a serial mediating role between psychological strain and suicidal ideation; third, depression mediated the association between psychological strain and suicidal ideation. However, the path through the sole mediation of hopelessness was not significant. In general, the results supported our four hypotheses, with the exception of hopelessness mediating the relationship between psychological strain and suicidal ideation.

As seen from Table 2, age was positively correlated with hopelessness and depression. That is perhaps because older athletes are more worried about their future career after retirement, as they may feel that they have invested too much on sports and do not have much opportunity to develop other life skills. The findings that female athletes demonstrated greater value strain and suicidal ideation supported the findings of Ren et al., who found that females reported greater value strain than males [44]. The results were also consistent with previous research where female athletes were demonstrated to be more prone to mental health problems [45]. The result that male athletes demonstrated greater hopelessness was supported by Lester’s finding that boys reported higher hopelessness than girls [46]. Elite athletes reported greater value strain, coping strain, suicidal ideation, and hopelessness. Elite athletes, who receive more attention, typically receive more negative social evaluations from the public, which may cause greater pressure, and eventually lead to mental health problems. Additionally, elite athletes are more likely to confront problems from training and competitions, such as injury, overtraining, and failure, which might lead to mental disorders [45,47]. Athletes playing non-confrontational sports demonstrated greater psychological strain, hopelessness, and depression. That is perhaps because it is more difficult to get direct and immediate feedback about their performance, which is crucial for mental health [48]. The results that athletes who were from urban areas and only-child families demonstrated greater hopelessness and depression supported the findings of Lee et al., who found that teenagers who were from urban area with no siblings had a higher tendency to have mental problems [49].

We found that psychological strain was related to suicidal ideation in athletes. This finding confirmed previous research; for example, a link between psychological strain and suicide was identified among populations with the same age as the athletes in this study, such as rural young people and college students [16,50]. Stewart et al. reported that athletes showed more improvement in negative affect (which was considered to be a risk factor for eating disorder) after participating in a cognitive dissonance-based program compared to a healthy weight intervention program [51]. Voelker et al. also reported the effectiveness of a cognitive dissonance-based program named “Bodies in Motion” in supporting positive body image in female collegiate athletes [52]. As psychological strain is considered to be much like cognitive dissonance, our finding also confirmed the results of Stewart et al. and Voelker et al. [51,52], which suggests that psychological strain theory could be applied to explain suicidal ideation among athletes. Due to the centralized training management pattern under the national competitive sports system, Chinese athletes grow up in a completely different environment from ordinary young people. However, we found that the results among athletes were consistent with those among others. Zhang et al. reported low to medium positive correlations (0.19–0.29) between psychological strain and suicidal ideation among college students who are in the same age as the athletes in this study but living in different environment [17]. In athletes, we found a similar degree of association (0.17), with no obvious difference from those among college students’ samples, suggesting that the strain theory is applicable to Chinese athletes.

Unexpectedly, we did not notice the mediating role of hopelessness between psychological strain and suicidal ideation in athletes. This result was not supported by previous studies, which revealed that hopelessness was a risk factor of suicidality [24,25,53]. This finding was not consistent with the findings of Zhang et al., who found that hopelessness mediated the effect of psychological strain on suicidal ideation among patients diagnosed with stomach cancer [28]. The current result was difficult to explain, but might be partly due to the following reasons. First, hopelessness was not as significant for athletes as for cancer patients in predicting suicidal ideation. Second, the impact of hopelessness on suicidal ideation was perhaps fully mediated by other factors (e.g., depression).

Indeed, we found a serial mediating effect of hopelessness and depression between psychological strain and suicidal ideation, and the path from hopelessness to suicidal ideation was fully mediated by depression. This finding, therefore, supported our hypothesis. The result that hopelessness had an impact on depression supported previous cross-sectional and longitudinal findings that hopelessness was associated with depression [33,34,35,36], and also supported the hopelessness theory of depression, which suggests that hopelessness is an immediate cause of depression [32]. Taken together, the present findings suggest that psychological strain is sequentially related to a sense of despair first, and then depression in athletes, which in turn contributes to suicidal ideation.

Additionally, depression was found to solely mediate the relationship between psychological strain and suicidal ideation. In other words, psychological strain also had an indirect effect via depression. This finding confirms previous research that found that depression was a risk factor of suicidality [29]. The result was also consistent with Zhang et al.’s recent finding that Chinese college students experiencing greater psychological strain demonstrated greater depression [54].

The present study has significant implications. First, to the authors’ knowledge, this study is the first to apply the psychological strain theory to explain suicidal ideation in Chinese athletes, and therefore provides a new theoretical basis for the study of mental health in athletes. Second, findings from this study provide further evidence for the importance of psychological strain, hopelessness, and depression in the explanation of suicidal ideation in athletes. Identifying the risk factors of suicidal ideation would help in the implementation of tailor-made preventive efforts accordingly [32]. Third, the results among athlete samples that confirmed the same results among other populations, such as college students, rural young people, and stomach cancer patients [16,17,28], provide more evidence to support the hypotheses of psychological strain theory, and expand the research scope of the strain theory. Additionally, our results included participants who were in two different developmental stages (adolescence and early adulthood). Adolescents are under the responsibility of their parents as socializing agents and are very influenced by peers [55,56]. By contrast, young adults are assuming some adult roles and have more responsibilities and duties [57,58]. Interestingly, although participants were in two different developmental stages, a common pattern about the relationship between psychological strain, suicidal ideation, hopelessness, and depression was found, so the findings of the present study are more generalizable in comparison with those obtained in a very limited age range. Last, the present study revealed that hopelessness and depression transmitted the influence of psychological strain on suicidal ideation, and, therefore, the study provides an in-depth elucidation of the specific psychological mechanisms by which psychological strain influences suicidal ideation.

Competitive sports put pressure on athletes, and the high demands and extensive training loads present potential threats to athletes’ mental health [1]. Studying the prevalence of suicidal ideation in athletes will help to draw the attention of academic, social, and government departments to the mental health problems of athletes. To improve an athlete’s competitive level and extend their sporting life, measures should be taken to help athletes avoid mental disorders, which is also conductive to the promotion of well-being and long-term development in general. Cognitive therapy approaches could be used to weaken or eliminate one or more conflicting stressors, by which we can reduce the intensity of psychological strain and provide early intervention and effective prevention strategies for athletes’ mental illness [59,60].

One limitation of the current study was that it was cross-sectional in nature, and thus, the causal relationship between variables could not be established. Second, convenient sampling was adopted; thus, the representativeness of the sample should be considered. Third, athletes in the present study were from national or provincial teams. Training and competitions constitute a major part of their lives. Therefore, the present findings may not be generalizable to amateur or non-professional athletes. Additionally, the present study was performed in a Chinese cultural setting, and the cross-cultural applicability of the conclusions requires further verification. Although the strain theory of suicide was reported to be applicable for both Chinese and American college student samples, the effects of psychological strain were demonstrated to have substantiated cross-cultural variations [17,61]. The Chinese centralized sports system is quite different with the sports system in other countries. It is unclear whether the structure of psychological strain is the same or different by cultural context. Future research could carry out cross-cultural research and compare the results obtained in this study with other groups of athletes from other nations—comparing the constructs of psychological strain and its impact on mental health [62]. Finally, the issue of suicide is complex. The study, treatment, and prevention of suicide cannot be reduced to one or two simplistic theories. The approach to this topic should be personalized, taking into account the personality, emotions, and psychotherapeutic dimensions as a whole.

## 5. Conclusions

This study sheds light on the important roles that psychological strain plays in elucidating mental health problems in athletes and demonstrates that the psychological strain model is suitable for explaining suicidal ideation among athletes. Psychological strain affects suicidal ideation not only directly but also indirectly through the mediation of hopelessness and depression. In athletes, psychological strain is sequentially associated first with a sense of hopelessness for the future and then depression, which is in turn related to suicidal ideation.

## Figures and Tables

**Table 1:** Descriptive statistics and correlation matrix for psychological strain, hopelessness, depression, and suicidal ideation in athletes.

	Mean	*SD*	1	2	3	4	5	6	7	8
1 Value strain	2.678	0.835	1.000	-	-	-	-	-	-	-
2 Aspiration strain	2.651	0.912	0.741 ***	1.000	-	-	-	-	-	-
3 Deprivation strain	2.400	0.944	0.611 ***	0.790 ***	1.000	-	-	-	-	-
4 Coping strain	2.252	0.854	0.620 ***	0.752 ***	0.729 ***	1.000	-	-	-	-
5 Psychological strain	2.479	0.794	0.805 ***	0.933 ***	0.902 ***	0.885 ***	1.000	-	-	-
6 Hopelessness	6.424	3.284	0.129 ***	0.224 ***	0.252 ***	0.250 ***	0.251 ***	1.000	-	-
7 Depression	15.671	9.239	0.302 **	0.400 ***	0.356 ***	0.407 ***	0.419 ***	0.614 ***	1.000	-
8 Suicidal ideation	0.176	0.381	0.165 ***	0.164 ***	0.083 *	0.202 ***	0.171 ***	0.100 **	0.238 ***	1.000

Notes: * *p* < 0.05; ** *p* < 0.01; *** *p* < 0.001.

**Table 2:** The associations of demographic variables with psychological strain, suicidal ideation, hopelessness, and depression.

Variables	Age *r*	Gender		Sports Level		Sports Event		Residence		Only Child	
		Male Mean (*SD*)	Female Mean (*SD*)	Non-Elite Mean (*SD*)	Elite Mean (*SD*)	Non-Confrontational Mean (*SD*)	Confrontational Mean (*SD*)	Urban Mean (*SD*)	Rural Mean (*SD*)	Yes Mean (*SD*)	No Mean (*SD*)
Psychological strain	0.027	2.462 (0.705)	2.505 (0.906)	2.438 (0.790)	2.523 (0.797)	2.541 * (0.697)	2.393 (0.909)	2.485 (0.737)	2.474 (0.852)	2.460 (0.696)	2.494 (0.861)
Value strain	−0.028	2.607 ** (0.745)	2.778 (0.940)	2.616 * (0.838)	2.742 (0.828)	2.751 ** (0.770)	2.573 (0.910)	2.688 (0.773)	2.667 (0.897)	2.625 (0.734)	2.717 (0.902)
Aspiration strain	0.038	2.652 (0.837)	2.649 (1.012)	2.588 (0.891)	2.716 (0.931)	2.706 * (0.817)	2.573 (1.029)	2.640 (0.849)	2.662 (0.976)	2.625 (0.823)	2.670 (0.974)
Deprivation strain	0.064	2.428 (0.852)	2.358 (1.061)	2.425 (0.949)	2.372 (0.939)	2.465 * (0.863)	2.306 (1.042)	2.401 (0.877)	2.397 (1.010)	2.417 (0.881)	2.386 (0.988)
Coping strain	0.002	2.206 (0.784)	2.317 (0.942)	2.176 * (0.839)	2.330 (0.864)	2.303 * (0.758)	2.179 (0.971)	2.269 (0.829)	2.233 (0.881)	2.226 (0.764)	2.271 (0.916)
Suicidal ideation	−0.058	0.132 ** (0.339)	0.238 (0.426)	0.104 *** (0.305)	0.251 (0.434)	0.194 (0.396)	0.150 (0.358)	0.193 (0.396)	0.157 (0.364)	0.166 (0.373)	0.183 (0.387)
Hopelessness	0.110 **	6.905 *** (3.462)	5.741 (2.882)	6.709 * (3.546)	6.127 (2.962)	6.793 *** (3.534)	5.900 (2.816)	6.759 ** (3.607)	6.069 (2.865)	7.039 *** (3.598)	5.964 (2.949)
Depression	0.096 **	15.703 (8.995)	15.625 (9.590)	15.681 (0.172)	15.660 (9.321)	16.780 * (9.418)	14.097 (8.755)	16.977 *** (9.492)	14.287 (8.767)	17.000 ** (9.533)	14.677 (8.895)

Notes: The associations of age with the variables were evaluated using Pearson correlations; the comparison of continuous and categorical variables between different demographic categories was evaluated using independent *t*-tests and chi-square tests, respectively; * *p* < 0.05; ** *p* < 0.01; *** *p* < 0.001.

**Table 3:** Risk of suicidal ideation among athletes by multivariate logistic regressions.

Variables		Model 1		Model 2		Model 3		Model 4	
		OR (95% CI)	*p*	OR (95% CI)	*p*	OR (95% CI)	*p*	OR (95% CI)	*p*
Age		0.941 (0.886–1.000)	0.052	0.933 (0.876–0.994)	0.033	0.927 (0.868–0.989)	0.022	0.913 (0.853–0.977)	0.008
Gender	Male	1.000	-	1.000	-	1.000	-	1.000	-
	Female	1.586 (1.064–2.365)	0.024	1.542 (1.029–2.313)	0.036	1.682 (1.111–2.547)	0.014	1.497 (0.979–2.289)	0.062
Level	Non-elite	1.000	-	1.000	-	1.000	-	1.000	-
	Elite	2.879 (1.894–4.377)	<0.001	2.889 (1.887–4.422)	<0.001	3.074 (2.001–4.722)	<0.001	3.122 (2.018–4.831)	<0.001
Sports Event	Non-confrontational	1.000	-	1.000	-	1.000	-	1.000	-
	Confrontational	0.800 (0.534–1.198)	0.278	0.847 (0.562–1.277)	0.428	0.880 (0.581–1.334)	0.548	0.897 (0.587–1.370)	0.614
Residence	Urban	1.000	-	1.000	-	1.000	-	1.000	-
	Rural	0.873 (0.572–1.332)	0.529	0.852 (0.554–1.308)	0.463	0.867 (0.561–1.341)	0.522	0.934 (0.600–1.454)	0.761
Only child	Yes	1.000	-	1.000	-	1.000	-	1.000	-
	No	1.109 (0.723–1.702)	0.634	1.079 (0.698–1.670)	0.731	1.133 (0.726–1.766)	0.583	1.157 (0.737–1.818)	0.526
Psychological strain		-	-	1.686 (1.334–2.132)	<0.001	1.553 (1.217–1.982)	<0.001	1.292 (0.991–1.685)	0.059
Hopelessness		-	-	-	-	1.102 (1.034–1.176)	0.0033	1.000 (0.925–1.080)	0.994
Depression		-	-	-	-	-	-	1.065 (1.036–1.095)	<0.001

**Table 4:** Path analysis.

Outcome Variables	Predictors	β	*SE*	95% CI	*p*
Hopelessness	Psychological strain	0.251	0.033	0.184–0.317	0.001
Depression	Psychological strain	0.282	0.026	0.231–0.331	0.001
	Hopelessness	0.543	0.024	0.493–0.590	0.001
Suicidal ideation	Psychological strain	0.087	0.038	0.011–0.164	0.026
	Depression	0.201	0.038	0.123–0.276	0.001

**Table 5:** Direct and indirect effects of psychological strain on suicidal ideation in athletes.

Effect Types	Mediators	Paths	Effect	*SE*	95% CI
Direct effect	-	Strain→Suicidal ideation	0.087 (50.88%)	0.038	0.011–0.164
Indirect effect	Hopelessness and depression	Strain→Hopelessness→ Depression→Suicidal ideation	0.028 (16.37%)	0.007	0.016–0.042
	Depression	Strain→Depression→Suicidal ideation	0.057 (33.33%)	0.012	0.035–0.082
Total indirect effect	-	-	0.084 (49.12%)	0.017	0.052–0.120
Total effect	-	-	0.171	0.035	0.106–0.243
